# 2022 Chinese expert consensus and guidelines on clinical management of toxicity in anti-CD19 chimeric antigen receptor T-cell therapy for B-cell non-Hodgkin lymphoma

**DOI:** 10.20892/j.issn.2095-3941.2022.0585

**Published:** 2023-03-02

**Authors:** Ping Li, Yang Liu, Yun Liang, Jian Bo, Sujun Gao, Yongxian Hu, Yu Hu, He Huang, Xiaojun Huang, Hongmei Jing, Xiaoyan Ke, Jianyong Li, Yuhua Li, Qifa Liu, Peihua Lu, Heng Mei, Ting Niu, Yongping Song, Yuqin Song, Liping Su, Sanfang Tu, Jianxiang Wang, Depei Wu, Zhao Wang, Kailin Xu, Zhitao Ying, Qingming Yang, Yajing Zhang, Fengxia Shi, Bin Zhang, Huilai Zhang, Xi Zhang, Mingfeng Zhao, Weili Zhao, Xiangyu Zhao, Liang Huang, Jun Zhu, Wenbin Qian, Weidong Han, Aibin Liang

**Affiliations:** 1 Department of Hematology, Shanghai Tongji Hospital, Tongji University School of Medicine, Shanghai 200065, China; 2 Department of Bio-therapeutic, Chinese PLA General Hospital, Beijing 100853, China; 3 Department of Hematology, The Second Affiliated Hospital, College of Medicine, Zhejiang University, Hangzhou 310009, China; 4 Department of Hematology, Chinese PLA General Hospital, Beijing 100853, China; 5 Department of Hematology, The First Hospital of Jilin University, Changchun 130012, China; 6 Center for Bone Marrow Transplantation, The First Affiliated Hospital, School of Medicine, Zhejiang University, Hangzhou 310058, China; 7 Institute of Hematology, Union Hospital of Tongji Medical College; Huazhong University of Science and Technology, Wuhan 430022, China; 8 Peking University People’s Hospital & Peking University Institute of Hematology, Beijing Key Laboratory of Hematopoietic Stem Cell Transplantation, Beijing 100044, China; 9 Department of Hematology, Lymphoma Research Center, Peking University Third Hospital, Beijing 100191, China; 10 Department of Hematology, The First Affiliated Hospital of Nanjing Medical University, Jiangsu Province Hospital, Nanjing 210029, China; 11 Department of Hematology, Zhujiang Hospital, Southern Medical University, Guangzhou 510280, China; 12 Department of Hematology, Nanfang Hospital, Southern Medical University, Guangzhou 510515, China; 13 Lu Daopei Institute of Hematology, Beijing 101102, China; 14 Department of Hematology, West China Hospital, Sichuan University, Chengdu 610041, China; 15 The Affiliated Cancer Hospital of Zhengzhou University, Zhengzhou 450008, China; 16 Department of Lymphoma, Key Laboratory of Carcinogenesis and Translational Research (Ministry of Education/Beijing), Peking University Cancer Hospital and Institute, Beijing 100142, China; 17 Department of Hematology, Shanxi Cancer Hospital, Taiyuan 030013, China; 18 State Key Laboratory of Experimental Hematology, National Clinical Research Center for Blood Diseases, Division of Pediatric Blood Disease Center, Institute of Hematology & Blood Disease Hospital, Chinese Academy of Medical Sciences & Peking Union Medical College, Tianjin 300020, China; 19 Department of Hematology, The First Affiliated Hospital of Soochow University, Jiangsu Institute of Hematology, National Clinical Research Center for Hematologic Diseases, Suzhou 215006, China; 20 Department of Hematology, Beijing Friendship Hospital, Capital Medical University, Beijing 100050, China; 21 Department of Hematology, The Affiliated Hospital of Xuzhou Medical University, Xuzhou 221006, China; 22 Institute of Blood and Marrow Transplantation, The Fifth Medical Center, Chinese PLA General Hospital, Beijing 100039, China; 23 Department of Lymphoma, Tianjin Medical University Cancer Institute & Hospital, National Clinical Research Center for Cancer, Key Laboratory of Cancer Prevention and Therapy, Tianjin, Tianjin’s Clinical Research Center for Cancer, Tianjin 300060, China; 24 Medical Center of Hematology, Xinqiao Hospital, State Key Laboratory of Trauma, Burn and Combined Injury, Army Medical University, Chongqing 400037, China; 25 Department of Hematology, Tianjin First Central Hospital, Tianjin 300192, China; 26 Department of Hematology, Shanghai Ruijin Hospital, Shanghai Jiao Tong University School of Medicine, Shanghai 200025, China; 27 Department of Hematology, Tongji Hospital of Tongji Medical College, Huazhong University of Science and Technology, Wuhan 430030, China

**Keywords:** CAR T-cell therapy, B-cell non-Hodgkin lymphoma, toxicity, cytokine-release syndrome, clinical management

## Abstract

Adoptive cellular immunotherapy with chimeric antigen receptor (CAR) T cells has emerged as a novel modality for treating relapsed and/or refractory B-cell non-Hodgkin lymphoma (B-NHL). With increasing approval of CAR T-cell products and advances in CAR T cell therapy, CAR T cells are expected to be used in a growing number of cases. However, CAR T-cell-associated toxicities can be severe or even fatal, thus compromising the survival benefit from this therapy. Standardizing and studying the clinical management of these toxicities are imperative. In contrast to other hematological malignancies, such as acute lymphoblastic leukemia and multiple myeloma, anti-CD19 CAR T-cell-associated toxicities in B-NHL have several distinctive features, most notably local cytokine-release syndrome (CRS). However, previously published guidelines have provided few specific recommendations for the grading and management of toxicities associated with CAR T-cell treatment for B-NHL. Consequently, we developed this consensus for the prevention, recognition, and management of these toxicities, on the basis of published literature regarding the management of anti-CD19 CAR T-cell-associated toxicities and the clinical experience of multiple Chinese institutions. This consensus refines a grading system and classification of CRS in B-NHL and corresponding measures for CRS management, and delineates comprehensive principles and exploratory recommendations for managing anti-CD19 CAR T-cell-associated toxicities in addition to CRS.

## Introduction

CAR T-cell therapy is a novel treatment approach for hematological malignancies^1–3^. Multiple CD19-targeted CAR T-cell products have been approved for the treatment of relapsed and/or refractory (r/r) B-cell non-Hodgkin lymphoma (B-NHL), including large B-cell lymphoma, follicular lymphoma, and mantle cell lymphoma, in the United States and Europe^4–7^. In China, 2 CAR T-cell products, axicabtagene ciloleucel^8^ and relmacabtagene autoleucel^9^, have been approved for use in adults with r/r large B-cell lymphoma. In addition, the number of registered clinical trials of CAR T cells in China has substantially increased in the past 5 years, and surpassed that in the United States. With the development of novel CAR T-cell products^10–12^ and innovative approaches, such as dual-target CAR T cells^13–15^ and sequential infusions of CAR T cells targeting different antigens^16–18^, CAR T-cell therapy is anticipated to be widely used to treat various tumors beyond B-NHL in the near future.

With increased understanding of CAR T-cell properties and experience in treating patients with CAR T cells, clinicians are becoming more concerned about potentially life-threatening CAR T-cell-associated toxicities^19–22^. CAR T cells show dynamic differences among hematological malignancies^23–26^, and CAR T-cell-associated toxicities in B-NHL have distinguishing features such as local cytokine-release syndrome (L-CRS)^27^. Nevertheless, previously published CRS grading and management guidelines have provided few specific recommendations for managing these toxicities^28–30^. This consensus aims to provide clinicians with a standardized guideline and recommendations for the diagnosis, prevention, and treatment of toxicities in patients with B-NHL treated with CAR T-cell therapy.

The contents of the consensus are divided into 7 sections: the first section includes a workup and risk assessment for CAR T-cell-associated toxicities at baseline, and the other 6 sections outline various toxicities, including CRS, CAR-T-cell-associated encephalopathy syndrome (CRES), hemophagocytic lymphohistiocytosis/macrophage activation syndrome (HLH/MAS), and other adverse events (AEs). Because CRS is the most commonly observed AE in CAR T-cell therapy, its clinical management is emphasized herein.

## Workup and risk assessment for toxicities at baseline

Essential workup procedures before CAR T-cell therapy should include a complete medical history and physical examination, with attention paid to node-bearing areas, evaluation of performance status, documentation of lymphoma classification and staging at diagnosis, previous treatments, and evaluation of opportunistic infections, as appropriate. Laboratory evaluations include complete blood count (CBC), urinalysis, blood chemistry profile, comprehensive metabolic panel, serum ferritin and lactate dehydrogenase, disseminated intravascular coagulation panel (including D-dimer, fibrinogen, prothrombin time, and partial thromboplastin time measurements), serum cytokine profile [including measurements for serum interleukin (IL) 6, tumor necrosis factor-α (TNF-α)], and C-reactive protein. Other recommended tests include hepatitis B/C virus, human immunodeficiency virus, and treponemia pallidum antibody evaluations. Epstein–Barr virus, cytomegalovirus and human herpes virus testing may be useful under certain circumstances. Imaging and other studies include chest/abdomen/pelvic computed tomography (CT) with contrast and/or whole body positron emission tomography/CT, brain magnetic resonance imaging (MRI), electrocardiography, and echocardiography. If any extranodal involvement, particularly in the bone marrow (BM), gastrointestinal (GI) tract, pleura/peritoneum, and central nervous system (CNS), is suspected, relevant examinations, including BM biopsy with aspirate, endoscopic ultrasound, and serosal and lumbar puncture to analyze serosal effusion and cerebrospinal fluid (CSF), should be performed. Organ function tests are recommended for patients with vital organ involvement. Optional workup procedures include BM cytogenetics (karyotype analysis) and pulmonary function tests.

The above workup is also designed to identify populations at higher risk of developing severe toxicities. These high-risk factors include the following: (1) ECOG ≥ 3^31^; (2) age ≥ 70 years; (3) high tumor burden, i.e., the sum of the product of the perpendicular diameters of multiple lesions (SPD) ≥ 100 cm^2 32^; (4) a bulky mass with diameter ≥ 10 cm; (5) a mass located in the pharynx or around the trachea, with compression symptoms; (6) a mass near luminal organs (e.g., the GI tract or bile duct), which may induce organ dysfunction because of tumor compression and infiltration; (7) serosal involvement with massive serous effusion (pleural/abdominal effusion); (8) hepatitis B antigen seropositivity, hepatitis B virus (HBV) DNA copy number above the upper limit of normal value, or active HBV infection without antiviral treatment; (9) involvement of vital organs (e.g., lung, kidney, or bone marrow); and (10) tumor-associated fever.

## CRS

CRS is defined as a supraphysiologic immune response after immunotherapy that leads to overactivation of endogenous or infused T cells and other immune effector cells^29,33,34^. The incidence rate of CRS among patients with B-NHL treated with CAR T cells is 23%–93%. Among these patients, 2%–22% experience severe CRS (sCRS, grade 3 or higher)^8,35,36^.

The mechanism of CRS remains elusive. Recent studies have indicated that activated monocytes/macrophages but not CAR T cells are major contributors to CRS^37,38^, and that endothelial activation and macrophage-released catecholamines may involve amplification of cytokine release and enhancement of inflammatory injury during CAR T-cell therapy^39,40^. Yuying Liu et al.^41^ have suggested that damage-associated molecular patterns released by pyroptotic cells may be upstream triggers of macrophage/macrophage activation. Direct contact between CAR T cells and macrophages through CD40-40 L^42^, CD69^43^, lymphocyte activation gene-3^44^, and TNF-α^45^ may also play important roles in this process.

### Classification and grading of CRS

Owing to its physiological features, CRS in B-NHL exhibits unique features^27,46^. Therefore, the classification and grading of CRS in B-NHL must be refined to help clinicians identify and manage this AE effectively.

**Figure 1:**
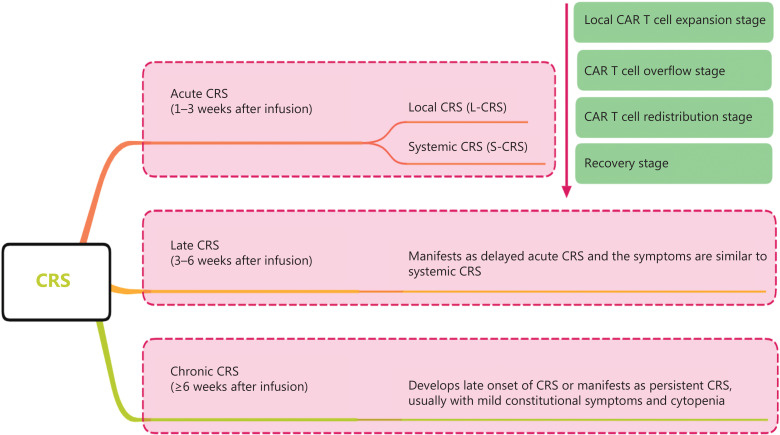
CRS staging and classification. CRS can be classified as acute CRS, late CRS, or chronic CRS according to onset time. CRS can also be classified on the basis of the location and site involved as local CRS and systemic CRS. CRS, cytokine-release syndrome; L-CRS, local cytokine-release syndrome; S-CRS, systemic cytokine-release syndrome; CAR, chimeric antigen receptor.

**Figure 2:**
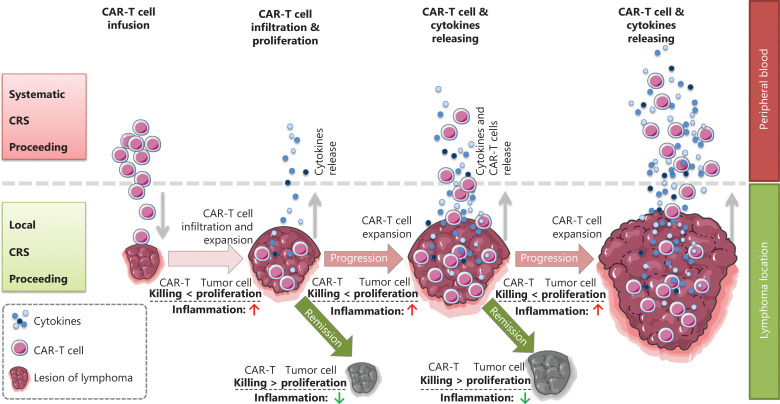
Schematic diagram of the *in vivo* distribution of CAR T cells. Schematic procedures from local to systemic CRS after CAR T cell infusion. During early stages after CAR T cell infusion, circulating CAR T cells delivered by intravenous infusion enter the target antigen-expressing tumor mass, thus resulting in CAR T cell expansion and simultaneous cytokine release within locally infiltrating sites, and tumor volume swelling due to local inflammatory reactions; this stage is referred to as clinically local CRS. In the later stage, cytotoxicity of CAR T cells leads to tumor collapse and volume shrinkage; subsequently, many activated CAR T cells and secreted cytokines enter the peripheral blood and trigger systemic CRS.

CRS can be classified as acute CRS (1–3 weeks after infusion), late CRS (3–6 weeks after infusion), and chronic CRS (≥ 6 weeks after infusion) according to the onset time. CRS can also be classified as L-CRS or systemic CRS (S-CRS) according to the location and site, involved ^27,47^ (**Figure 1**). During early stages of CAR T-cell therapy, the infused CAR T cells accumulate in the tumor, expand locally, and release several cytokines that in turn trigger a local inflammatory response, which is defined as L-CRS. Subsequently, the locally expanded CAR T cells and cytokines “overflow” into the circulatory system, thereby promoting S-CRS (**Figure 2**).

L-CRS is frequently observed in patients with a high tumor burden or bulky mass, and it manifests as redness/swelling/enlargement and exudation/effusion in or around the lesion, or even perforation or bleeding at the lesion site. Unlike those of L-CRS, the clinical manifestations of S-CRS are characterized by fever, hypotension, and hypoxia, with or without organ dysfunction^27,47–49^. Given that the previously published CRS grading systems are based only on the severity of S-CRS symptoms, which may not be fully applicable to B-NHL, we propose a new CRS grading system that incorporates the specific manifestations of L-CRS^29^. The grades are defined by the presence of fever (≥38 °C), the severity of hemodynamic compromise, the severity of hypoxia and the severity of local inflammatory manifestations of lesions surrounding tissues and organs (**Table 1**).

**Table 1:** Grading of CRS in CAR T-cell treatment for B-NHL

Symptom or sign of CRS	Grade 1	Grade 2	Grade 3	Grade 4
Fever (temperature ≥ 38 °C)	Yes	Yes	Yes	Yes
And				
Hypotension (systolic blood pressure < 90 mmHg)	No	Vasopressors not required	Response to one type of vasopressor	Multiple vasopressors required
And/or				
Hypoxia (oxygen requirement: SaO_2_ > 90%)	No	Low-flow* nasal oxygen required	High-flow* oxygen through a nasal cannula, a mask, a nonrebreather mask, or a venturi mask^‡^ required	Positive pressure mechanical ventilation (noninvasive mechanical ventilation, or tracheal intubation and mechanical ventilation) required
And/or				
Inflammatory manifestations of lesions, surrounding tissues, and organs	No	Enlarged lesion not causing compression symptoms or affecting functions of organs around the tumor	Enlarged lesion resulting in compression symptoms or serous effusion in tissues around the tumor, etc.; potentially compensation of functions of adjacent organs	Enlarged lesion resulting in compression symptoms, edema/bleeding/perforation in the surrounding tissues, or massive serous effusion, etc.; decompensated functions of surrounding organs

CRS, cytokine-release syndrome; CAR, chimeric antigen receptor; B-NHL, B-cell non-Hodgkin lymphoma; SaO_2_, arterial oxygen saturation. *Low flow is defined as ≤ 6 L/min, and high flow is defined as > 6 L/min. ^‡^Venturi mask: a mask manufactured based on the Venturi effect. As oxygen flows into the mask through the narrow pores, a negative pressure is created surrounding the jet airflow, thus resulting in air flowing into the mask from the open edge; the size of the edge determines the ratio of oxygen to air.

### Monitoring and management of acute CRS

The peak window of CRS risk is typically the first 3 weeks after CAR T-cell infusion, which is a critical period for CRS management^30^. Thus, clinicians must closely monitor patients to ensure early diagnosis and prompt intervention for CRS. The workup includes a complete physical examination, with special attention to vital signs and lesion sites, assessment of organ systems, and laboratory tests^19,50^. Electrocardiographic monitoring is recommended from the time of CAR T-cell infusion until acute CRS is relieved. For patients with high risk of sCRS, this monitoring should continue for at least 3 weeks or until the high-risk factors are resolved. Laboratory tests include CBC, complete metabolic panel, blood chemistry profile, coagulation profile, serum ferritin, lactate dehydrogenase, serum cytokine profile, and CAR transgene copies (or CAR T-cell percentage in peripheral blood mononuclear cells), which must be rechecked at least 3 times per week for 2 weeks postinfusion, once per week for the next 2 weeks, and every 3 months thereafter. For patients with high sCRS risk, the above tests should be performed more frequently or even daily. Imaging studies to assess the lesions and involved organs may be considered for select patients.

Management of CRS in B-NHL should be performed in line with the grade of this toxicity, as shown in **Table 2**. If CRS symptoms persist or become aggravated for more than 24 h, a management strategy for a higher CRS grade should be initiated immediately. Patient transfer to the intensive care unit (ICU) is recommended when sCRS develops. In general, patients with CRS are administered a combination of tocilizumab and corticosteroids, in addition to supportive care. For prolonged S-CRS or substantial symptoms or comorbidities, treatment with tocilizumab at 4–8 mg/kg (not exceeding 800 mg) is advised, and administration can be repeated after 6 h, as needed. Furthermore, the use of anti-TNF-α agents can be considered if L-CRS is present or for persistent and refractory fever. For patients with grade 4 or persistent CRS despite anti-IL-6 and/or anti-TNF-α therapy, the use of corticosteroids may be considered. For patients with high risk of sCRS, β-receptor blockers should be administered from the day of CAR T-cell infusion^40^. Plasma exchange can be performed to treat emergent CRS. Additional supportive care includes antipyretic measures, intravenous fluid boluses, and/or vasopressors for patients with hypotension, and/or supplemental oxygen for those with hypoxia.

**Table 2:** Clinical management of CRS in CAR T-cell treatment of B-NHL^29,30^

CRS grade	Patient monitoring	Supportive care	Cytokine antagonists	Corticosteroids	Plasmapheresis
Grade 1	Assess vital signs at least 3 times daily	Acetaminophen and a cooling blanket are the first choice for treatment of fever Ibuprofen can be a second choice for treatment of fever, if not contraindicated Blood cultures, urinalysis, urine cultures, and chest radiography should be ordered for assessment of infections Start empiric broad-spectrum antibiotics and G-CSF for patients with neutropenia Maintain IV fluids to keep patients well-hydrated Provide symptomatic management of organ toxicities	IL-6R antagonists can be considered prophylaxis (recommended)	Not recommended	Not recommended
Grade 2	Assess vital signs with continuous electrocardiogram monitoring	Provide IV fluid bolus as needed If hypotension persists after 2 fluid boluses and anti-IL-6 therapy, start vasopressors, consider ICU transfer, perform echocardiography, and initiate other methods of hemodynamic monitoring Manage fever and constitutional symptoms as in grade 1 Provide supplemental oxygen Perform symptomatic management of organ toxicities	Use one type of cytokine antagonist Recommended antibodies include: IL-6R antagonist (recommended) TNF-α antibody (exploratory recommendation) TNF-α receptor antibody (exploratory recommendation)	For persistent refractory symptoms after one type of cytokine antagonist therapy, consider: Dexamethasone 10 mg IV q6h (recommended)	Not recommended
Grade 3	Assess vital signs with continuous electrocardiogram monitoring Consider transferring the patient to the ICU for further monitoring and treatment	Provide IV fluid boluses and vasopressors as needed Transfer to the ICU, and perform echocardiography and hemodynamic monitoring Manage fever and constitutional symptoms as indicated for grade 1 CRS Provide supplemental oxygen, including high-flow oxygen delivery and noninvasive positive pressure ventilation Perform symptomatic management of organ toxicities	Use 2 or 3 types of cytokine antagonists together. Recommended antibodies include: IL-6R antagonist (recommended) TNF-α antibody (exploratory recommendation) TNF-α receptor antibody (exploratory recommendation)	For persistent refractory symptoms after 2 or 3 types of cytokine antagonist therapy, consider: Dexamethasone 10–20 mg IV q6h (recommended)	If cytokine therapy is ineffective or corticosteroid is contraindicated, order plasmapheresis evaluation of blood transfusion (exploratory recommendation)
Grade 4	Assess vital signs with continuous electrocardiogram monitoring ICU transfer for further monitoring and treatment is recommended	Provide IV fluids and vasopressors, and perform hemodynamic monitoring Manage fever and constitutional symptoms as in grade 1 CRS Provide mechanical ventilation Perform symptomatic management of organ toxicities	Use 3 types of cytokine antagonists together. Recommended antibodies include: IL-6R antagonist (recommended) TNF-α antibody (exploratory recommendation) TNF-α receptor antibody (exploratory recommendation)	Consider: Dexamethasone 20 mg IV q6h (recommended) Methylprednisolone IV 1 g/day (recommended)	Order plasmapheresis after evaluation of blood transfusion (recommended)

CRS, cytokine-release syndrome; CAR, chimeric antigen receptor; B-NHL, B-cell non-Hodgkin lymphoma; G-CSF, granulocyte colony-stimulating; IV, intravenous; IL-6R, interleukin 6 receptor; ICU, intensive care unit; TNF-α, tumor necrosis factor-α.

Severe CRS is typically associated with dysfunction in multiple vital organs, such as the heart, liver, and kidney, which can be fatal and should be thoroughly evaluated and properly managed. Of note, L-CRS may cause specific organ damage, depending on the location of the lesion^4,51^. When L-CRS occurs, patients with GI involvement or lesions around the GI tract may experience mucosal damage or even vascular rupture and bleeding at lesion sites^52^. Thus, tissue damage may provide more possibilities for the intestinal flora to promote S-CRS; consequently, gut purging and oral antibiotics to inhibit the intestinal flora before CAR T-cell therapy are recommended^53^. Prophylactic use of anti-TNF-α agents may also be considered when compression symptoms occur in patients with a mass around the trachea or serosal involvement, and anti-TNF-α therapy and local intervention, such as tracheotomy and drainage of serous effusion, should be performed. The heart is another organ requiring special attention^54,55^: cardiac function should be closely monitored when the tumor lesion is located around the heart^56–58^. Bridging therapy before CAR T-cell therapy is necessary for patients with this underlying risk. Detailed clinical management procedures for AEs in organs during L-CRS are shown in **Table 3**.

**Table 3:** Management of AEs in involved organs during L-CRS in CAR T-cell treatment of B-NHL

Symptom	Management
Lung involvement	Use IL-6R antagonist (such as tocilizumab 4–8 mg/kg IV) (recommended)
Massive abdominal lesions*	Begin infection control (include protective isolation, food and drink disinfection, etc.) according to the “transplant protocol”, even during conditioning therapy (exploratory recommendation) Adjust the intestinal flora (for example, oral administration of intestinal probiotics such as Clostridium butyricum Enterococcus triple viable tablets) (exploratory recommendation) Administer TNF-α antibody as prophylaxis on day 3 and day 5 after CAR T-cell infusion (exploratory recommendation) For grade 2–3 L-CRS, provide antibody-combination therapy for mainly blocking TNF-α pathway (exploratory recommendation)
Massive serous effusion due to involvement of serous cavity mass	Drain paracentesis fluid before CAR T-cell infusion (exploratory recommendation) Indwell catheter of the serous cavity until CRS is relieved (exploratory recommendation) Inject tocilizumab (80 mg) into the serous cavity 3–5 days before CAR T-cell infusion (exploratory recommendation)
Heart involvement	Obtain evaluation of potential adverse events (dysrhythmia, heart failure, myocardial damage, etc.) by a cardiologist (exploratory recommendation) CAR T-cell therapy is recommended only after heart lesions are resolved (exploratory recommendation)
Involvement of skin, muscle, and connective tissue	Before CAR T-cell therapy, decrease or eliminate lesions of skin and soft tissues (exploratory recommendation) Enhance the prevention of local skin infections (local medication, debridement, etc.) (exploratory recommendation) Administer empirical anti-infection therapy early after CAR T-cell infusion (exploratory recommendation)
Involvement of central nervous system	Obtain evaluation by a neurology specialist (exploratory recommendation) Use CAR T-cell therapy with caution in cases of unclear efficacy and high risks (exploratory recommendation)
Dysphagia due to compression of neck lesions	Indwell a nasogastric tube for feeding to prevent aspiration (recommended) Provide bridging therapy or intensive conditioning therapy to relieve compression symptoms before CAR T-cell infusion (exploratory recommendation)
Dyspnea caused by compression of neck lesions	Indwell a nasogastric tube for feeding to prevent aspiration (recommended) Provide bridging therapy or intensive conditioning therapy to alleviate compression symptoms before CAR T-cell infusion (exploratory recommendation) Formulate an emergency plan for tracheal intubation and provide bedside tracheostomy devices (exploratory recommendation)

AEs, adverse events; L-CRS, local cytokine-release syndrome; CAR, chimeric antigen receptor; B-NHL, B-cell non-Hodgkin lymphoma; IL-6R, interleukin 6 receptor; IV, intravenous; etc., et cetera; TNF-α, tumor necrosis factor-α. *Massive abdominal lesions: maximum tumor diameter ≥ 10 cm.

### Late CRS

Late CRS is generally considered delayed acute CRS, typically occurring within 3–6 weeks after CAR T-cell infusion^59^. Similarly to those of S-CRS, the clinical manifestations of late CRS are fever, cytopenia, elevated transaminase levels, coagulation disorders, increased CAR copy number, and residual tumors. Late CRS should be distinguished from lymphodepletion therapy-associated hematological toxicities, GI toxicities, and infections. The monitoring and management strategies for acute CRS are similar to those for late CRS.

### Chronic CRS

Chronic CRS is defined as inflammation- or CAR T-cell-associated AEs that occur after 6 weeks of CAR T-cell infusion^29,60,61^. The clinical manifestations of chronic CRS include (1) intermittent low-grade fever (below 38 °C); (2) fatigue and anepithymia; (3) cytopenia, particularly thrombocytopenia; (4) CAR T-cell re-expansion in the peripheral blood; (5) residual tumors; and (6) interstitial pneumonitis or bronchiectasis-like manifestations in some patients. Chronic CRS should be distinguished from infections and hematological toxicity^59,62^. Management of chronic CRS includes anti-TNF-α agents for treatment of pulmonary symptoms and supportive care^63^. CBC should be monitored closely, and component blood transfusion can be performed for supportive care in select patients.

## CAR T-cell-associated encephalopathy syndrome

CRES is another common AE that occurs with CAR T-cell therapy and is characterized by a pathological CNS process after therapy^29,64,65^. Although the pathophysiological mechanism of CRES remains unclear, its severity has been shown to be associated with high levels of serum cytokines, high tumor burden, disruption of the blood brain barrier, CAR structure, and brain mural cells expressing CD19^66,67^.

According to published data, the incidence of CRES is 20%–64%, and 5%–28% of cases in patients with B-NHL who receive CAR T-cell therapy are of grade 3 or higher^28,35,36,68–70^. CRES typically occurs within 8 weeks after CAR T-cell infusion, with a median duration of 4–6 days. Neurological symptoms of CRES include headache, agitation, cognitive dysfunction, tremors, ataxia, language disturbance, somnolence, papilloedema, and seizures^30,71,72^, and secondary cerebral edema is a leading cause of death^47,73^.

Distinguishing CRES from cerebrovascular events such as cerebral hemorrhage and infarction is crucial, because thrombocytopenia and coagulation disorders frequently occur after CAR T-cell therapy^74^. Medical history inquiry and brain MRI examination may be useful. Single epileptic seizures often occur in some patients after CAR T-cell therapy. Clinicians should be cautious when diagnosing CRES, particularly in patients with a history of cerebrovascular accidents and seizures in this context.

According to the CAR-T-cell-therapy-associated toxicity (CARTOX) CRES grading scale^30^, grade is defined by the CARTOX-10 score, elevated intracranial pressure, and seizures/motor weakness. Workup for grading CRES includes a thorough neurological evaluation through fundoscopic examination and electroencephalography (EEG), neuroimaging assessment through MRI or CT scans, and measurement of CSF pressure^30^. Similarly to CRS, CRES management should be based on the appropriate toxicity grade, as shown in **Table 4**. In addition to supportive care, corticosteroids are the cornerstone of the management strategy for CRES. However, tocilizumab is not recommended for treating CRES unless CRS is concurrent. ICU monitoring is recommended for patients with grade 3 or 4 CRES. For patients with a history of seizures or a high risk of CRES, prophylactic use of levetiracetam can be implemented before CAR T-cell therapy^73,75^.

**Table 4:** Clinical management recommendations for CRES^30^

CRES grade	Symptom or sign	Clinical management recommendations for CRES
Grade 1	CARTOX-10 score*: 7–9	Provide supportive therapy, aspiration precautions, and IV hydration. Withhold oral intake of food, medicine, and fluids during assessment of swallowing ability.
	CSF pressure: NA^‡^	If swallowing is impaired, convert all oral medications and/or nutrition to IV. Avoid medications that may cause CNS depression.
	Seizures or motor weakness: NA^‡^	Under careful monitoring, low doses of lorazepam (0.25–0.5 mg IV q8h) or haloperidol (0.5 mg IV q6h) can be used in patients with agitation. Obtain an expert consultation in neurology. Perform funduscopic examination to evaluate papilloedema. CNS MRI with or without contrast; diagnostic CSF puncture with measurement of the pressure in the CSF; spine MRI (if the patient has focal peripheral neuropathy); and CT scan of the brain (if a CNS MRI cannot be performed) are all options. The patient should undergo 30 min of EEG examination every day until symptoms disappear if conditions permit; if no seizures are detected on EEG, the patient should be continually prescribed levetiracetam (p.o., 750 mg) q12 h. If the EEG reveals nonconvulsive status epilepticus, consult a specialist or follow treatment recommendations (assess airway, breathing, and circulation; check blood glucose). The patient should be prescribed levetiracetam and lorazepam; levetiracetam 500 mg IV bolus, as well as maintenance doses of lorazepam 0.5 mg IV, with additional 0.5 mg IV every 5 min, as needed, up to a total of 2 mg to control electrographic seizures. If seizures persist, transfer the patient to the ICU and treat with a phenobarbital loading dose of 60 mg IV. Maintenance doses after resolution of nonconvulsive status epilepticus are as follows: lorazepam (0.5 mg IV q8h for 3 doses); levetiracetam (1,000 mg IV q12h); phenobarbital (30 mg IV q12h). In the event of concurrent CRS, consider tocilizumab (8 mg/kg, IV) or siltuximab (11 mg/kg, IV).
Grade 2	CARTOX-10 score: 3–6	Continue supportive therapy and neurological evaluation, as described for grade 1 CRES. In cases of concurrent CRS, strengthen the management of CRS (see above).
	CSF pressure: NA^‡^	Dexamethasone (10 mg, IV, q6h) or methylprednisolone (1 mg/kg, IV, q12h) should be administered if there is no response to anti-IL-6 therapy or no concurrent CRS.
	Seizures or motor weakness: NA^‡^	Consider transferring the patient to the ICU if CRES is associated with grade ≥ 2 CRS.
Grade 3	CARTOX-10 score: 0–2	Continue supportive therapy and neurological evaluation as described for grade 1 CRES. Referral to the ICU is recommended.
	CSF pressure: stage 1 or 2 papilloedema, or CSF pressure < 20 mmHg	Anti-IL-6 therapy is recommended in the case of concurrent CRS, if it has not been administered previously, as described for grade 2 CRS. In the event that anti-IL-6 therapy does not produce a response, consider glucocorticoids. Corticosteroids are recommended until the CRES resolves to grade 1, and the dose can be gradually decreased in the absence of concurrent CRS.
	Seizures or motor weakness: partial seizure, or nonconvulsive seizures on EEG in response to benzodiazepine	For CSF pressure 20 mmHg with stage 1 or 2 papilloedema, manage in accordance with the recommendations [acetazolamide 1,000 mg IV, followed by 250–1,000 mg IV q12h (adjust dose on the basis of renal function and acid–base balance, monitored 1 or 2 times daily)]. Consider repeating neuroimaging (CT or MRI) every 2–3 days if the patient continues to experience grade ≥ 3 CRES.
Grade 4	CARTOX-10 score: the patient is critically ill and/or with obtundation, and the assessment tasks cannot be performed	Continue supportive therapy and neurological evaluation as described for grade 1 CRES. Perform ICU monitoring, taking into account mechanical ventilation. In the case of grade 3 CRES, provide anti-IL-6 therapy and repeat neuroimaging as described.
	CSF pressure: stage 1 or 2 papilloedema, or CSF pressure ≥ 20 mmHg, or manifestations of brain edema	Administer high-dose corticosteroids until the CRES resolves to grade 1, then decrease the dose. For example, methylprednisolone (ivgtt, 1 g/day) can be administered for 3 consecutive days, followed by rapid tapering at 250 mg q12h for 2 days, 125 mg q12h for 2 days, and 60 mg q12h for 2 days. Convulsive status epilepticus should be treated in the department of neurology or as recommended by the physician.
	Seizures or motor weakness: generalized seizures, convulsive or nonconvulsive status epilepticus, or new motor weakness	Papilloedema of stage ≥ 3 with CSF pressure ≥ 20 mmHg or brain edema should be treated by the department of neurology or as recommended.

The doses indicated for all medications are for adults. Tocilizumab has a maximum cumulative dose of 800 mg. CRES, CAR T-cell-associated encephalopathy syndrome; CARTOX-10, CAR T-cell therapy-associated toxicity-10 points; CSF, cerebrospinal fluid; IV: intravenous; CNS, central nervous system; MRI, magnetic resonance imaging; CT, computed tomography; EEG: electroencephalogram; CRS, cytokine-release syndrome; ICU: intensive-care unit; ivgtt: intravenous guttae. *In the CARTOX 10, one point is assigned for each of the following tasks that is performed correctly (normal cognitive function is defined by an overall score of 10): orientation to year, month, city, hospital, and president/prime minister of country of residence (total of 5 points for directional positioning description test); name 3 objects around (for example, indicate key, shoe, hat; maximum of 3 points for naming test); write a standard sentence, for example, “China’s national flag is a five-star red flag” (total of 1 point for concentration test); count backward from 100 in tens, for example, 100, 90, 80.. 20, 10 (total of 1 point for concentration test). ^‡^NA, not applicable.

## Hemophagocytic lymphohistiocytosis/macrophage activation syndrome

HLH/MAS are a group of severe immunological diseases characterized by excessive activation of T cells and macrophages, lymphohistiocytic tissue infiltration, and immune-mediated multiorgan dysfunction^76–78^. CAR T-cell-associated HLH/MAS usually occurs during the recovery phase of CRS or accompanies CRS^29,78,79^. Compared with S-CRS, HLH/MAS has characteristic clinical manifestations that include hepatosplenomegaly; hemophagocytes and hemophagocytosis in the bone marrow; cytopenia in at least 2 hematopoietic cell lineages; hypertriglyceridemia; and elevated serum levels of ferritin, cytokines, and soluble CD25^80–82^. HLH/MAS may result in prolonged leukopenia or neutropenia, thus increasing the risk of serious infection. The early phase of HLH/MAS is easily overlooked, because the features also frequently occur in CRS, infection, and bone marrow suppression after CAR T-cell therapy^29,30^. HLH/MAS should be suspected in the presence of the following conditions^78,80–84^: high levels of blood CAR T cells for more than 2 weeks and again after 2 weeks; recurrent fever with cytopenia or hepatosplenomegaly; and sustained increases in serum ferritin. To date, standard clinical management of HLH/MAS in patients treated with CAR T-cell therapy is not well established. According to cumulative experience from multiple domestic centers and consultation with experts with experience in treating this toxicity, we provide the following recommendations for clinical management of HLH/MAS^81–84^: (1) closely monitor vital signs and CBC in patients after CAR T-cell therapy; (2) closely monitor serum concentrations of ferritin and triglyceride; (3) HLH/MAS should be suspected if a patient has unexplained fever and cytopenia after CAR T-cell infusion; (4) if HLH/MAS is diagnosed, additional therapy with low-dose etoposide (50–100 mg/m^2^ per week) is recommended; (5) JAK-2 inhibitors (ruxolitinib), CTLA-4 agonists (abatacept), and anti-CD52 antibodies (alemtuzumab) can be used for select patients; (6) plasma exchange may be considered in emergencies; and (7) use of novel agents, such as anti-interferon-γ antibody, may be considered under certain circumstances.

## Bone marrow suppression

Cytopenia caused by bone marrow suppression is one of the most common AEs in patients after CAR T-cell therapy^70,85–87^; the incidence of grade 3–4 neutropenia, anemia, and thrombocytopenia is 45%–69%, 30%–37%, and 15%–30%, respectively^9,35,88^. At least 15% of patients experience prolonged severe cytopenia beyond 3 months^85,86^.

Given that cytopenia increases risks of infection and relevant complications (e.g., cerebral hemorrhage), the following management measures should be performed according to its grade: (1) prophylaxis for bacterial and fungal infections until severe leukopenia or neutropenia resolves; (2) supplemental low-flow oxygen and packed red blood cell transfusion for patients with severe anemia; (3) prophylactic use of hemostatic agents and platelet transfusion for patients with severe thrombocytopenia; and (4) additional preventive measures (e.g., room disinfection by ultraviolet light or use of a soft toothbrush) for patients with any grade of leukopenia or thrombocytopenia.

## Infection

Infections after CAR T-cell therapy are common and are reported in as many as 70% of patients^9,36,59,89^; as many as 40% of these infections occur within the first month^79^. The most common infections are bacterial (16%–30%), and these usually occur within the first 2 weeks. Factors that may contribute to infection include lymphodepletion or prior chemotherapy, CAR T-cell-mediated B-cell aplasia/hypogammaglobulinemia, and prolonged cytopenia.

In such cases, the manifestations of infections can mimic CRS in terms of fever, hypotension, and hypoxia, thus posing a major diagnostic dilemma^29,30,90,91^. Nonetheless, no biomarkers are available to differentiate the 2 disorders. Moreover, because infections usually occur in the context of CRS and may exacerbate CRS, early identification and timely intervention are crucial. One previous study has indicated that the distinct signature for severe infections includes a double peak of serum IL-6 levels, and high serum levels of IL-8 and IL-1β, which may aid in differential diagnosis between severe infection and CRS^51,79^. If CRS and infection are not distinguishable, we recommend that the combination of CRS treatment and antimicrobial prophylaxis be considered.

During CAR T-cell therapy, concurrent infections may complicate CAR T-cell-associated toxicities, including CRS and neurotoxicity. Moreover, approximately 10% of severe or life-threatening infections may occur during and after the lymphodepletion period. For patients with active or uncontrolled infection at baseline, CAR T-cell therapy cannot be initiated until the infection is resolved^21,70^. Prophylaxis against *Pneumocystis jirovecii* pneumonia and zoster reactivation is generally applied before lymphodepletion therapy and for 3 months thereafter^51,92–94^. Of note, CAR-T therapy may pose a risk of HBV reactivation in carriers. For HBV carriers, prophylactic use of entecavir should be initiated before lymphodepletion therapy and continue for at least 6 months. Monthly follow-up to monitor HBV DNA levels is also necessary for early detection of HBV reactivation^95,96^. The decision to provide prophylaxis against bacterial or fungal infections should be risk-adjusted on the basis of patient characteristics, such as prior hematopoietic stem cell transplantation and infection history. After lymphodepletion chemotherapy, the use of granulocyte colony-stimulating factor^97^ can be considered when neutrophil counts decrease to < 0.5 × 10^9^/L. If infection is suspected, empiric broad-spectrum antibacterial therapy should be initiated immediately^98^. Empiric antifungal therapy is usually added after 4–7 days in patients expected to have persistent neutropenia.

Infections that occur at specific sites (skin and soft tissue, urinary system, and catheter-associated infections) are more common with CAR T-cell-mediated systemic immunosuppression, in contrast to other antitumor therapies. Of note, infections of the skin and soft tissue or serosal involvement by tumors may be severe and aggravated by concurrent L-CRS. The management principles for special-site infections are provided in **Table 5**.

**Table 5:** Management of infections at specific sites

Infection site	Treatment
Skin and soft tissues	Provide effective wound care (e.g., eradication of necrotic tissue) Perform biopsy of skin lesions for histology and culture Administer empiric antibiotics administered to the wound Refer to the principal treatment of L-CRS for high-risk patients with severe infection caused by L-CRS
Urinary system	Monitor routine urine Replace urinary catheters regularly Collect microbiological samples If the patient develops infection-associated symptoms, initiate empiric antibiotic treatment according to local protocols
Intravenous catheter	Perform blood cultures for patients suspected of having CRBSI before administration of antimicrobial therapy Remove the catheter from patients with persistent hemodynamic instability or severe sepsis as soon as possible After organism and antibiotic sensitivities have been identified, the antibiotic regimen should be tailored on the basis of culture results
Serosal cavity catheter	Perform routine maintenance measures to prevent infection include daily disinfection of the exit site If the patient develops infection-associated symptoms, administer anti-infection treatment

L-CRS, local cytokine-release syndrome; CRBSI, catheter-associated bloodstream infections.

## Other toxicities

### B-cell aplasia/hypogammaglobulinemia

Prolonged B-cell aplasia/hypogammaglobulinemia, an anticipated on-target, off-tumor toxicity, is a characteristic adverse effect after CAR T-cell therapy^51,99^ that can be diagnosed according to absolute B-cell counts < 61 cells/μL and serum immunoglobulin G (IgG) ≤ 400 mg/dL^100^. Almost all patients who receive CAR T-cell therapy experience varying degrees of B-cell deficiency^36,70^, thus increasing the risk of humoral immunity dysfunction-associated infections. Prophylactic intravenous injection of human immunoglobulin (IVIG) is a routine adjuvant treatment for patients who receive CAR T-cell therapy. The practical algorithm for management of hypogammaglobulinemia is as follows^93^:

- Replacement with monthly IVIG until recovery of B cells to the normal range or 6 months after CAR T-cell infusion.
- For patients with serum IgG ≤ 400 mg/dL and serious or recurrent infection, monthly IVIG replacement should continue until risk factors are eliminated.
- Regular monitoring of serum levels of IgG, IgM and IgA and blood CD19+ or CD20+ B-cell counts is recommended for patients at high risk of infection.

### Tumor lysis syndromes

Tumor lysis syndromes (TLS) are a group of disorders caused by rapid release of large amounts of intracellular contents and metabolites into the systemic circulation^101,102^. Clinical manifestations of TLS include hyperuricemia, hyperphosphatemia, hypocalcemia, hyperkalemia, and acute renal insufficiency. Diagnostic criteria for TLS include (1) a 25% increase in the levels of serum potassium; (2) a 25% decrease in calcium levels; (3) serum creatinine > 221 μmol/L; (4) 25% increase in the levels of serum uric acid or urea nitrogen; and (5) cardiac arrhythmia and renal failure in some patients^103,104^.

TLS prophylaxis should be considered for patients with high tumor burden (SPD ≥ 100 cm^2^ or bulky mass with diameter ≥ 10 cm) or high tumor proliferation activity (Ki67 ≥ 85%)^101,102^. We recommend that hydration and alkalization be initiated 24 h before lymphodepletion therapy, and if necessary, diuretics be used to ensure a urine volume > 3,000 mL/day. Sodium bicarbonate may be administered to maintain urine pH in 7.0–7.5.

The foundation of TLS treatment includes (1) frequent monitoring of electrocardiography, blood pressure, and oxygen saturation; (2) adequate intravenous hydration (≥3,000 mL/day) to maintain urine volume (≥3,000 mL/day), with diuretics used if necessary; (3) sodium bicarbonate administered to ensure urine pH between 7.0 and 7.5; (4) correction of fluid and electrolyte disturbances; (5) allopurinol or febuxostat for treatment of hyperuricemia; and (6) hemodialysis for patients who develop renal failure with uncorrected electrolyte disorder^101,102,104,105^.

### Hypersensitivity reactions

The incidence of infusion-associated allergic reactions is relatively low (up to 3%), and allergic shock rarely occurs. In general, allergic reactions can be easily mistaken for a secondary symptom of other AEs. For example, skin rash usually occurs within the first 2 weeks after CAR T-cell infusion and resolves spontaneously within 3–5 days. In addition to allergies, skin rash may be attributable to CRS-induced increased fragility of the capillary endothelium. The population at high risk of developing allergic reactions includes those who may be hypersensitive to (1) *in vitro* culture reagents during CAR T production, (2) viral vector impurity, and (3) T-cell activation resulting from a pre-CAR T unresolved inflammatory background.

Management principles include the following: (1) exclusion of patients with hypersensitivity at enrollment; (2) strict control of the procedure and reagents during CAR T-cell manufacturing; (3) antimicrobial therapy before infusion to eliminate an *in vivo* inflammatory background; and (4) prophylactic use of anti-allergic agents (e.g., diphenhydramine or promethazine)^106^.

### Excessive CAR T-cell proliferation

CAR T-cell levels should be monitored within the first month after CAR T-cell infusion. Blood samples should be collected once every 2–3 days within the first 2 weeks and once per week thereafter. Excessive CAR T-cell proliferation should be highly suspected under the following conditions^107^: (1) white blood cell counts in peripheral blood ≥ 10 × 10^9^/L; (2) lymphocyte percentage in white blood cells ≥ 70%; and (3) absolute CAR+ T-cell counts > 600 cells/μL. Indications that should be considered for identifying this condition include the following:

1. Is the expansion of CAR T cells consistent with the changes in tumor size?
2. Do CAR T cells localize to or expand at other sites in additional to the peripheral blood?
3. Is the uncontrolled proliferation of activated T cells caused by viral infection?

Management practices to control excessive CAR T-cell proliferation include glucocorticoids and other immunosuppressive agents (e.g., anti-thymocyte globulin or anti-CD52 antibodies). A combination of 2 or more immunosuppressive agents may be used in severe cases.

### Secondary malignancies

On the basis of previous studies, the incidence of secondary malignancies after CAR T-cell therapy is approximately 15%^59,108^, a percentage similar to that of patients receiving other antitumor therapies^109–113^. Acute myeloid leukemia and myelodysplastic syndromes are the most commonly observed secondary hematologic malignancies^28,59,108^. Secondary malignancies usually develop after the first year of CAR T-cell therapy^114,115^, and regular follow-up is important for early recognition of these disorders after CAR T-cell therapy. Some studies have suggested that combination therapy with epigenetic drugs, such as decitabine, may improve patient prognosis.

## Conclusions

CAR T-cell therapy is a potentially curative treatment for patients with r/r B-NHL. However, CAR T-cell-associated toxicities may decrease the survival benefit from CAR T-cell therapy. To date, a thorough recommendation for management of CAR T-associated toxicities in B-NHL is lacking. In response to this need, we compiled this consensus on the basis of our current understanding and clinical experience from multiple domestic institutions. The consensus refines the CRS grading system and classification and provides comprehensive and detailed practice recommendations for grading, monitoring, and managing toxicities observed in CAR T-cell therapy for B-NHL. We hope that the consensus will be useful for CAR T-cell therapy for B-NHL by helping clinicians effectively manage CAR T-cell-associated toxicities.

The consensus has several limitations. First, most of the evidence provided was derived from clinical studies on CAR T cells, mostly targeting CD19, yet toxicity profiles and features can vary across CAR T-cell products and individuals. Second, some exploratory recommendations proposed herein are based on case/case series reports or clinical experience, without sufficient evidence. Future multicenter prospective clinical trials and real-world studies are needed to develop more appropriate toxicity mitigation and optimization strategies.
